# Age-Related and Region-Specific Morphological and Molecular Alterations of Astrocytes and Microglia Following Chronic Oxidative Stress in Male Wistar Rats

**DOI:** 10.3390/ijms27219817

**Published:** 2026-11-01

**Authors:** Alexandra V. Sentyabreva, Andrey D. Gerasimov, Ekaterina A. Melnikova, Chulpan B. Kharisova, Gleb M. Aliper, Anna M. Kosyreva

**Affiliations:** Petrovsky National Research Center of Surgery, 117418 Moscow, Russia

**Keywords:** neurodegeneration, oxidative stress, microglia, astrocytes, inflammaging

## Abstract

Aging is associated with chronic pro-inflammatory background and increased risk of susceptibility to oxidative stress and neurodegenerative processes. However, age-dependent morphological and molecular changes in glial cells under chronic oxidative stress remain insufficiently characterized. This study aimed to investigate age- and region-specific alterations in astrocytes and microglia in the hippocampus and the entorhinal cortex (EntC) of adult (4–6 months) and old (22–24 months) male Wistar rats following chronic AlCl_3_ administration (100 mg/kg/day for 60 days). Spatial learning and memory were assessed using the Morris water maze. GFAP+ astrocytes and Iba1+ microglia were evaluated by immunohistochemistry and morphometry. Hippocampal 4-HNE and pTau (Ser404) were assessed by ELISA and Western blotting, respectively. Hippocampal expression of *Tnf*, *Tgfb*, *Mmp9*, *Bdnf*, *Ntrk2*, and *Gpx4* was evaluated by RT-qPCR, while serum MMP-9 and HSP-70 concentrations were measured by ELISA. Aging was associated with increased glial cell size in both brain regions, elevated hippocampal *Tnf* expression, decreased *Tgfb* and *Ntrk2* expression, and increased serum MMP-9 and HSP-70 serum concentrations. In adult rats, AlCl_3_ exposure was accompanied by changes in astrocytic morphology and increased Iba1+ cell density in the EntC, whereas molecular compensatory mechanisms remained preserved in the hippocampus. Only old AlCl_3_-treated rats demonstrated reduced success in locating the former platform position in the Morris water maze and significantly elevated hippocampal 4-HNE levels, consistent with increased lipid peroxidation. Although hippocampal pTau (Ser404) immunoreactivity showed an age-related increase, no statistically significant effect of AlCl_3_ exposure or age + AlCl_3_ was detected. In this group, AlCl_3_ exposure was associated with increased Iba1+ cell density in both regions, hypertrophic astrocytic morphology in the hippocampus, and reduced astrocytic area with fragmented processes in the EntC. These changes coincided with decreased hippocampal *Bdnf* and *Gpx4* expression, as well as co-existence of reduced serum MMP-9 and HSP-70 concentrations. Overall, these findings demonstrate that aging influences region-specific glial morphological alterations and associated molecular responses to chronic AlCl_3_-induced oxidative stress. This model demonstrated a translational approach to provide insights regarding mechanisms of age-related neurodegeneration.

## 1. Introduction

Senescent cells accumulate during aging; these cells are characterized by the secretory phenotype associated with senescence (SASP) [1]. As a result of their secretory activity, inflammaging develops—a systemic, aseptic, persistent, and low-level pro-inflammatory background [2]. Inflammaging is one of the key risk factors for the development of age-related diseases, including neurodegenerative ones leading to dementia [3], which is one of the most common causes of disability and mortality worldwide [4]. It is a syndrome and the outcome of various diseases; however, most often dementia manifests in the late stages of Alzheimer’s disease (AD) [5]. AD remains a major social and medical problem, as approaches to early, effective, and accessible diagnosis and treatment have not yet been developed.

This is partly due to the fact that the pathogenesis of AD has not yet been sufficiently studied. More than 90% of experimental studies on modeling neurodegeneration and developing treatment approaches are conducted using various transgenic rodent lines or via intracerebral administration of amyloid [6]. Such studies are relevant only for early-onset familial AD (FAD), which accounts for less than 5% of AD cases. Furthermore, almost all studies are conducted on adult animals, whereas studies of age-related neurodegeneration are more appropriately conducted on old animals. However, older rodents do not develop human-specific diseases such as atherosclerosis, type 2 diabetes, and AD [7]. Consequently, a relevant stimulus is required to initiate these conditions—for example, oxidative stress caused by an excess of reactive oxygen species (ROS). Although ROS are not the only or primary culprit of an initiation of neurodegenerative processes and AD in particular, it could be a valuable translational approach if modeled in old animals.

One of the ROS inducers used to model AD is AlCl_3_. Exposure to it results in behavioral abnormalities, pro-inflammatory responses, and biochemical and histopathological changes in various brain structures [8,9,10,11,12,13]. However, these results were obtained only in adult animals. In recent years, the involvement of astrocytes and microglia in the development of neurodegenerative processes has been actively studied. Like any other cells, they undergo senescence [14]. Being immune cells, microglia are the first to respond to an excess of pro-inflammatory factors, including ROS. As a result of activation, they intensify the pro-inflammatory environment in the brain, which negatively affects astrocyte function, also leading to their activation and a reduction in neuroprotection [15]. It has previously been shown that in vitro incubation of microglial cells with AlCl_3_ led in their sustained activation followed by dysfunction [16,17], which is also a characteristic of aging and AD-like neurodegeneration, which we observed in old rats in our previously published study [18]. However, the effect of oxidative stress induced by long-term AlCl_3_ administration on astrocytes and the resulting morphological and molecular changes in old animals has not been studied, as well as region-specific changes in microglia and astrocytes in this model.

This study was designed as an exploratory investigation aimed at characterizing age-dependent and region-specific patterns of glial responses to chronic AlCl_3_-induced oxidative stress. This approach was chosen because the morphological and molecular responses of astrocytes and microglia to prolonged oxidative stress in aged animals, particularly their regional differences between the hippocampus and entorhinal cortex (EntC), remain insufficiently characterized. The selection of the regions studied was based on their sequential involvement in the pathological process. In human, the EntC was assessed as a site of primary vulnerability (early stages), while the hippocampus was identified as a major limbic region involved following early entorhinal alterations [19,20]. This experimental approach allows the investigation of age- and region-specific responses to chronic oxidative stress, which may be relevant to mechanisms implicated in neurodegenerative disorders. Accordingly, the study integrated several complementary levels of analysis, including glial cell morphology and morphometry, expression of selected inflammation-, neuroprotection-, and antioxidant defense-related genes, and circulating markers of inflammation and cellular stress response.

## 2. Results

### 2.1. Behavioral Changes Were Observed Due to Aging and Oxidative Stress

When assessing memory and spatial orientation functions in the Morris water maze, adult rats in both the control and AlCl_3_-treated groups demonstrated effective learning, quickly locating the platform on both the first and second days of training (Figure 1A,B). By the end of the first day, the median time required to locate the platform was 8 s for the control group and 12 s for the experimental group; by the end of the second day, these times were 5 s and 4 s, respectively. Old rats in both groups required more time for searching and orientation, particularly when the starting point from which they were released into the pool was changed (Figure 1A,B). On the first day of training, the median time to locate the platform was 29 s for the control group and 60 s for the experimental group. By the end of the second day of training, older rats in both groups were successful in locating the platform; the median time to locate the platform from the final (8th) starting point was 15 s for the old control rats and 17 s for the old AlCl_3_-treated animals.

During the probe trial without the platform, conducted 7 days after the second day of training, the following results were observed: all 10 adult control rats crossed the former platform location (100%), as well as all 10 adult AlCl_3_-treated animals (100%); 9 out of 10 old control rats crossed the former platform location (90%), whereas only 4 out of 10 old AlCl_3_-treated rats did (40%) (Figure 1C). Pairwise comparisons using Fisher’s exact test with Benjamini–Hochberg correction revealed a significantly lower success rate in old AlCl_3_-treated rats compared with both adult AlCl_3_-treated rats (adjusted *p* = 0.026) and old control animals (*p* = 0.043). Animals that did not reach the target location during the observation period (60 s) were treated as right-censored observations. Kaplan–Meier analysis revealed significant overall differences among the experimental groups (log-rank test, χ^2^(3) = 17.485, *p* < 0.001). Pairwise log-rank comparisons with Benjamini–Hochberg correction demonstrated a significant difference between adult and old AlCl_3_-treated rats (adjusted *p* = 0.024), whereas no significant differences were detected between the other groups (Figure 1D). No differences were observed among all the groups when comparing the number of crossings over the former platform location (Figure 1E).

### 2.2. Levels of 4-HNE and pTau Were Altered Due to Oxidative Stress

To assess lipid peroxidation and explore potential changes in tau-related molecular alterations, hippocampal 4-HNE concentrations and GAPDH-normalized pTau (Ser404) immunoreactivity were measured. Two-factor analysis revealed a significant main effect of AlCl_3_ exposure on hippocampal 4-HNE levels (H = 6.566, *p* = 0.010, η^2^H = 0.410), whereas neither the effect of age (H = 0.393, *p* = 0.531) nor the age × AlCl_3_ interaction (H = 0.093, *p* = 0.760) was significant. At the same time, there was a difference between old control and AlCl_3_-treated animals—4-HNE concentration in the hippocampus was higher in AlCl_3_-treated rats (*p* = 0.039). All other groups did not differ among each other despite the tendency to increasing of 4-HNE concentration in the hippocampus of adult AlCl_3_-treated rats (Figure 2A).

Two-factor analysis revealed a significant main effect of age (H = 9.845, *p* = 0.0017), whereas neither AlCl_3_ exposure (H = 1.927, *p* = 0.165) nor the age × AlCl_3_ interaction (H = 0.195, *p* = 0.659) was statistically significant. Dunn’s post hoc test with Benjamini–Hochberg correction demonstrated significantly higher values in old AlCl_3_-treated rats compared with adult AlCl_3_-treated rats (*p* = 0.030). No statistically significant differences were detected between the other groups (Figure 2B,C).

### 2.3. Morphology and Numbers of Microglia and Astrocytes Changed Region-Specifically Due to Aging and Oxidative Stress

To assess changes in the morphological properties of astrocytes and microglia during aging and AlCl_3_ exposure a morphological and morphometric study of Iba1+ and GFAP+ cells was conducted.

IF staining of microglia and astrocytes in the hippocampus of both groups of adult animals revealed small, moderately branched cells (length 15–25 µm). In older animals of the control group, microglial cells were characterized by increased size (length >30 µm), as described previously in more detail [21]. At the same time, in old AlCl_3_-treated animals, an increase in cell size and process thickness was also observed in astrocytes (Figure 3A–D).

In the hippocampus, adult control rats showed a median density of 8.7 Iba1+ cells/mm^2^ and a median microglial cell area of 73.35 µm^2^ (Figure 4A,E,F). In old controls, microglial density did not differ significantly from that in adult controls (13.3 cells/mm^2^), whereas cell area was significantly increased to 153.8 µm^2^ (Figure 4C,E,F). AlCl_3_ exposure did not significantly affect either microglial density or cell area in adult rats (Figure 4B,E,F). Old AlCl_3_-treated rats showed a 2.3-fold increase in Iba1+ cell density relative to old controls and 1,7-fold increase relative to adult AlCl_3_-treated rats, while microglial cell area was only increased compared with adult AlCl_3_-treated animals (Figure 4D–F). Two-factor analysis revealed a significant main effect of age and AlCl_3_ exposure on the number of Iba1+ microglial cells, as well as age effect on the Iba1+ cells area in the hippocampus.

In the EntC, adult control rats had a median Iba1+ cell density of 6.5 cells/mm^2^ and a median cell area of 73.84 µm^2^ (Figure 5A,E,F). In old controls, cell density remained unchanged (6.7 cells/mm^2^), whereas microglial cell area increased markedly to 130.6 µm^2^ (Figure 5C,E,F). In adult AlCl_3_-treated rats, Iba1+ cell density was significantly higher than in adult controls with no significant change in cell area (Figure 5B,E,F). In old AlCl_3_-treated rats, microglial density increased 2.8-fold relative to old controls, while cell area was greater than in adult AlCl_3_-treated rats (Figure 5D–F). There was a significant main effect of AlCl_3_ exposure on the number of Iba1+ microglial cells, while age effect was large on the Iba1+ cells area in the EntC.

In the hippocampus, adult control rats showed small (approximately 15–20 μm), moderately branched GFAP+ astrocytes (Figure 6A), whereas AlCl_3_ exposure increased process branching without altering cell size (Figure 6B). In old controls, astrocytes were enlarged (>20–30 μm) and displayed numerous thin processes (Figure 6C); in old AlCl_3_-treated rats, they remained enlarged and exhibited hypertrophied, highly branched processes (Figure 6D). GFAP+ cell numbers did not differ significantly between adult control and AlCl_3_-treated rats or between adult and old controls, although a downward trend was observed in adult AlCl_3_-treated animals (Figure 6E). Astrocyte area increased with age and AlCl_3_ exposure, but did not differ significantly between old control and old AlCl_3_-treated rats (Figure 6F). A significant size effect of age as well as age × AlCl_3_ interaction was revealed on the area of GFAP+ astrocytes, but not on their numbers.

In the EntC, adult control astrocytes were likewise small and moderately branched (Figure 7A), while AlCl_3_ treatment increased arborization without affecting cell size (Figure 7B). Old control astrocytes were enlarged and characterized by fewer, thickened processes (Figure 7C), whereas old AlCl_3_-treated cells showed thinned and fragmented ones (Figure 7D). GFAP+ cell numbers did not differ among groups (Figure 7E), whereas cell area increased from 52.1 µm^2^ in adult controls to 113.5 µm^2^ in old controls and 112.3 µm^2^ in adult AlCl_3_-treated rats. In old AlCl_3_-treated rats, astrocyte area decreased significantly to 77.3 µm^2^ compared with both old controls and adult AlCl_3_-treated animals (Figure 6F). No significant size effect on GFAP+ astrocytes numbers was observed, while there was a pronounced age × AlCl_3_ interaction for their area.

The size effect characteristics identified during the morphometric analysis are summarized in Table 1.

### 2.4. Expression of Inflammaging and Neuroprotection Markers in the Hippocampus Changed Differently Due to Aging and Oxidative Stress

To assess the molecular and genetic changes in the hippocampus resulting from aging and against the background of oxidative stress induced by AlCl_3_ intake, we investigated the expression of pro- and anti-inflammatory cytokines and markers of neuroprotection.

Examining the expression of the pro-inflammatory cytokine *Tnf* in the hippocampus of old rats, there were significant differences between adult and old control (*p* = 0.0096) and AlCl_3_-treated rats (*p* = 0.0096). There were no differences within pairs of adult and old animals. A large age-associated effect was observed (H = 16.651, *p* < 0.001, η^2^H = 0.595). Neither the main effect of AlCl_3_ exposure (H = 0.273, *p* = 0.601, η^2^H = 0.010) nor the age × AlCl_3_ interaction (H = 0.004, *p* = 0.951, η^2^H < 0.001) was statistically significant (Figure 8A). The opposite tendency was observed in expression of anti-inflammatory cytokine *Tgfb* with same significant main effect of age on *Tgfb* expression (H = 15.537, *p* < 0.001, η^2^H = 0.486) and insignificant size effect of AlCl_3_ exposure (H = 0.457, *p* = 0.499, η^2^H = 0.014) or the age × AlCl_3_ interaction (H = 0.051, *p* = 0.822, η^2^H = 0.002). Dunn’s post hoc test demonstrated significant differences between adult and old control rats (*p* = 0.011) and adult and old AlCl_3_-treated rats (*p* = 0.011). No significant differences were observed between control and AlCl_3_-treated animals within either the adult or old groups (Figure 8B).

In the hippocampus, the expression level of *Mmp9*, a marker of both SASP and microglial activation. *Mmp9* expression was significantly higher in adult AlCl_3_-treated rats compared with adult controls (*p* = 0.028) and old AlCl_3_-treated rats (*p* = 0.016). No significant differences were detected between other groups. Two-factor analysis displayed a significant age × AlCl_3_ interaction (H = 9.005, *p* = 0.0027, η^2^H = 0.265), indicating a large size effect. Neither the main effect of age (H = 1.081, *p* = 0.298, η^2^H = 0.032) nor AlCl_3_ exposure (H = 0.043, *p* = 0.836, η^2^H = 0.001) was statistically significant (Figure 8C).

The expression of *Bdnf*, encoding a key neurotrophic factor produced predominantly by neurons but also by glial cells, including astrocytes and microglia. Dunn’s post hoc test revealed significantly lower *Bdnf* expression in old AlCl_3_-treated rats compared with old control rats (*p* = 0.036) and adult AlCl_3_-treated rats (*p* = 0.038). No significant differences were observed between adult control and adult AlCl_3_-treated rats or between adult and old control animals. Two-factor analysis revealed significant main effects of age (H = 4.558, *p* = 0.033, η^2^H = 0.134) and AlCl_3_ exposure (H = 5.894, *p* = 0.015, η^2^H = 0.173) on *Bdnf* expression. In contrast, the age × AlCl_3_ interaction was not statistically significant and showed a small effect size (H = 0.942, *p* = 0.332, η^2^H = 0.028) (Figure 8D). *Ntrk2* encoded BDNF receptor TrkB, which is primarily present on neurons and astrocytes. There was a significantly lower *Ntrk2* expression in old AlCl_3_-treated rats compared with adult AlCl_3_-treated rats (*p* < 0.001). The age-related difference between adult and old control animals did not remain statistically significant after correction (pBH = 0.089). No significant effect of AlCl_3_ exposure was detected within either adult or old animals. Effect size analysis indicated a pronounced effect of age on *Ntrk2* expression (H = 17.928, *p* < 0.001, η^2^H = 0.512), corresponding to a large effect. In contrast, the main effect of AlCl_3_ exposure was not statistically significant and was characterized by a small effect size (H = 0.705, *p* = 0.401, η^2^H = 0.020). Similarly, the age × AlCl_3_ interaction was not statistically significant and showed a small effect size (H = 0.906, *p* = 0.341, η^2^H = 0.026) (Figure 8E). Also, significantly lower *Gpx4* expression was observed in old AlCl_3_-treated rats compared with old controls (*p* = 0.044) and adult AlCl_3_-treated rats (*p* < 0.001). No significant differences were observed between adult control and adult AlCl_3_-treated rats or between adult and old control animals. A pronounced main effect of age was detected in expression of *Gpx4* (H = 13.610, *p* < 0.001, η^2^H = 0.454) as well as age × AlCl_3_ interaction (H = 3.850, *p* = 0.050, η^2^H = 0.128). The main effect of AlCl_3_ exposure was not statistically significant (H = 1.825, *p* = 0.177, η^2^H = 0.061) (Figure 8F).

### 2.5. Protein Levels of Inflammation and Stress Response Markers in Blood Serum Associated with Glial Changes Due to Aging and Oxidative Stress

To assess adaptive capacity during aging at the systemic level, as well as following AlCl_3_ administration, the levels of MMP-9 and the heat shock protein chaperone HSP-70 in blood serum were measured using an ELISA.

MMP-9 in blood serum was significantly higher in old control rats compared with adult controls (Z = −3.663, pBH = 0.001), as well as between old control and old AlCl_3_-treated animals (*p* = 0.05). No significant differences were observed between adult control and adult AlCl_3_-treated rats (*p* = 0.138) or between adult and old AlCl_3_-treated animals (*p* = 0.996). A significant main effect of age (H = 5.984, *p* = 0.014, η^2^H = 0.187) and age × AlCl_3_ interaction (H = 7.432, *p* = 0.006, η^2^H = 0.232) were revealed, while the main effect of AlCl_3_ exposure was not statistically significant (H = 0.033, *p* = 0.856, η^2^H = 0.001) (Figure 9A).

HSP-70 is a protein belonging to the chaperone family, whose function is to reorganize misfolded proteins or initiate their degradation if reassembly into a functional state is impossible [22]. It was observed that HSP-70 concentration was higher in adult AlCl_3_-treated rats compared with adult controls (*p* = 0.029), but old AlCl_3_-treated rats exhibited significantly lower HSP-70 concentrations than old controls (*p* = 0.005). In addition, serum HSP-70 differed significantly between adult and old control animals (*p* = 0.005) and between adult and old AlCl_3_-treated animals (*p* = 0.038). Separately, age (H = 0.431, *p* = 0.511, η^2^H = 0.012) and AlCl_3_ exposure (H = 0.487, *p* = 0.485, η^2^H = 0.013) did not has a pronounced size effect for serum HSP-70 concentration, whereas age × AlCl_3_ interaction indicated a large interaction effect (H = 13.762, *p* < 0.001, η^2^H = 0.372) (Figure 9B).

## 3. Discussion

Conventional strains rodents, such as Wistar, do not develop most age-related diseases characteristic of humans, including neurodegenerative diseases [7]. However, their cells are also susceptible to senescence, which inevitably leads to inflammaging. The development of a pro-inflammatory state resulting from these processes is associated with systemic depletion of compensatory resources. This also applies to brain cells and their ability to adapt to adverse effects, such as oxidative stress. The brain is particularly susceptible to oxidative damage because of its high oxygen consumption, abundant content of polyunsaturated fatty acids (PUFAs), and relatively high levels of redox-active transition metal ions. Brain phospholipids are enriched in PUFAs, particularly arachidonic acid (AA) and docosahexaenoic acid (DHA), which are highly vulnerable to reactive oxygen species because of their multiple double bonds. Oxidative degradation of PUFAs initiates lipid peroxidation and generates a range of reactive aldehydic products, including 4-hydroxy-2-nonenal (4-HNE) [23]. Numerous studies have demonstrated increased levels of lipid peroxidation products including 4-HNE in different brain regions in AD patients as well as patience with mild cognitive impairment which precedes the clinical manifestation of AD [24,25,26]. Proteins modified by these reactive products have been detected predominantly in AD-vulnerable regions. Also it was shown that increasing 4-HNE level led to inhibited tau dephosphorylation [25,27]. Within affected regions, 4-HNE and other lipid peroxidation-derived adducts have been localized mainly to neuronal cytoplasm and neurofibrillary tangles [27], supporting a close association between oxidative lipid damage and AD-related neurodegeneration. In the present study, chronic AlCl_3_ exposure was associated with increased hippocampal 4-HNE levels, indicating enhanced lipid peroxidation. Western blot analysis also revealed a significant age-related difference in GAPDH-normalized pTau (Ser404) immunoreactivity. However, the present findings do not establish AlCl_3_-induced tau hyperphosphorylation. Nevertheless, the observed age-related differences in pTau (Ser404) increase warrant further investigation using larger cohorts, total Tau normalization, and antibodies targeting additional phosphorylation sites, including Ser396, Ser202/Thr205, and Thr231. Moreover, behavioral assessment revealed age-dependent differences in spatial learning and memory following chronic AlCl_3_ exposure. Although old AlCl_3_-treated rats retained the ability to locate the hidden platform by the end of training, they demonstrated reduced success in identifying its former location during the probe trial conducted seven days later. This finding may indicate impaired long-term spatial memory retention or retrieval, as delayed probe trials are commonly used to assess spatial reference memory [28]. Previous studies have also demonstrated that prolonged AlCl_3_ exposure can affect spatial learning and memory in rats [29], potentially in association with oxidative stress-related alterations. The observed pattern might partly resemble certain aspects of mild cognitive impairment characterized by measurable cognitive decline that precedes the severe functional deterioration associated with dementia [30].

Compared to adult animals, the absolute number of microglial cells in the EntC and hippocampus did not change with aging. According to the literature, the number of microglia cells can vary significantly depending on the age of the animals and the brain region. However, it has been shown that the number of microglia in the neocortex and the hippocampus is fairly stable in adult and old rats in the absence of any external influences [31,32], which is consistent with our findings. In the brains of old rats, microglia underwent somatic hypertrophy in both the cortex and hippocampus. Their cell bodies and processes became significantly larger and more swollen compared to those of adult control rats. This increase in soma area corresponded with a classic hallmark of age-related microglial “priming” or senescence [33], although this parameter alone does not unequivocally proved microglial priming or cellular senescence. The morphological characteristics of Iba1+ microglia in AlCl_3_-treated adult rats did not change. However, morphometric analysis revealed that in the EntC of these animals, median number of Iba1+ was 2.5-fold relative to the control group. At the same time, no statistically significant differences in the density of microglial cells were found between the control and AlCl_3_-treated adult rats in the hippocampus. According to the literature, hippocampal microglia are characterized by more complex morphology and phagocytic activity compared to those in the cerebral cortex in mice [34,35]. Presumably, these features might be associated with higher activity of antioxidant enzymes (glutathione peroxidase, catalase, superoxide dismutase, etc.) in the hippocampus than in the cortex in male Wistar rats, regardless of age [36]. Consequently, it might be assumed that although the dose and duration of AlCl_3_ administration used in this study stimulated ROS production, the antioxidant defense system in the hippocampus compensated for their effects. This was confirmed by the preserved *Gpx4* expression pattern. As a result, sustained activation led to increased Iba1+ cells number did not occur in the hippocampus in AlCl_3_-treated adult animals, indicating a high adaptation threshold of this brain region to oxidative stress. Morphological signs of Iba1+ microglial cells alteration were detected only in AlCl_3_-treated old rats. In these cells, there was an enlarged cell body area relative to adult rats but shrunken compared to older control animals alongside fragmentation of processes and deramification without hypertrophy, as shown previously [21]. These features might resemble some morphological characteristics described in dystrophic microglia under pathological conditions. If the pro-inflammatory state in the CNS is severe or highly prolonged—for example, in advanced age or in neurodegenerative diseases such as AD—microglia may become overwhelmed and lose their structural integrity in similar way [37,38]. However, because microglial functional activity, senescence markers, and detailed three-dimensional process architecture were not assessed, these observations should be interpreted as structural alterations rather than definitive evidence of microglial dystrophy or functional impairment.

Metabolic homeostasis in the brain depends on close integration between neurons and glial cells, primarily astrocytes [39]. Astrocytes support the functioning of neuronal mitochondria by ensuring ATP production, lipid biogenesis, regulation of calcium metabolism, and the removal of excess ROS [40]. Substrates such as lactate, provided by astrocytes, are important precursors for ATP production in neurons [40]. In addition, astrocytes play a pivotal role in modulating oxidative stress in neurons by supplying them with glutathione and glutamine as a substrate for glutamate production, which is necessary for glutathione synthesis [41]. Senescent astrocytes become reactive and can simultaneously produce SASP components and reduce the synthesis of metabolites essential for neurons [41,42]. Morphological analysis revealed increased astrocyte cell size and processes excessive branching and hypertrophy in the EntC and the hippocampus of old control rats—features commonly associated with astrocytic reactivity and aging-related morphological alterations [43]. The reduction in the number of processes and their thickening in GFAP+ astrocytes in the EntC might indicate their reactivity [43], which is closely associated with more intense oxidative stress in this region. This correlates with changes in astrocyte morphology in patients with AD [44], as well as with a more pronounced IHC reaction with pTau in the EntC of older rats in the experimental group, as we previously demonstrated [18]. In AlCl_3_-treated adult rats, morphological changes in GFAP+ astrocytes were observed in both the hippocampus and the entorhinal cortex due to their hyper-ramification. Apparently, the accumulation of ^27^Al in these region, which we demonstrated previously [18], and the resulting increase in pro-inflammatory background are sufficient for GFAP+ astrocytes in adult rats to acquire an altered phenotype, but insufficient to induce proliferation and pronounced morphological changes of microglial cells. This highlights the vulnerability of astrocytes to oxidative stress as an important aspect of their role in establishing a pro-inflammatory background in the brain and presumably in the reduction in neuroprotective functions. In the hippocampus of old AlCl_3_-treated rats, GFAP+ astrocytes exhibited enlarged cell bodies and more extensively branched processes; however, no statistically significant increase in GFAP+ cell density was detected compared with old controls. In contrast, GFAP+ astrocytes in the EntC exhibited a reduction in cell area and alterations in process morphology, including thinner and less extensive processes, consistent with a region-specific morphological response to oxidative stress corresponding to atrophy. Such state of astrocytes possibly could be one of the early signs of AD [45] and, like their altered morphofunctional state, is closely associated with the initiation and progression of neurodegenerative processes. According to the literature, senescence and atrophy in astrocytes result in a reduction in both the accumulation and synthesis of glutamate and its transport from astrocytes to neurons [45,46]. This leads to a disruption of long-term potentiation [47], which reduces the efficiency of neuronal interactions and impairs the maintenance of synaptic connections between them. Thus, gradual suppression of neuronal function occurs, leading subsequently to the development of neurodegeneration. Astrocytic alterations, including atrophy, might contribute to neurodegenerative processes by compromising neuronal support, particularly in brain regions vulnerable to oxidative stress [43]. It also resulted in more intense hyperphosphorylation of the tau protein [48], which is consistent with our previously published data [18]. Such morphological changes in microglia and astrocytes have not been previously described in AlCl_3_-induced neurodegeneration in old rats with manifest inflammaging. This provides a deeper understanding and improves in vivo modeling of age-related neurodegeneration.

A decrease in *Bdnf* expression was observed in the hippocampus only in AlCl_3_-treated old rats. We previously demonstrated a decrease in *Bdnf* expression in the prefrontal cortex also exclusively in this group [18]. Although neurons are considered a major source of BDNF in the adult brain, astrocytes can also produce and release BDNF, particularly in response to injury and inflammatory or metabolic stress. Therefore, the reduced *Bdnf* expression observed in old AlCl_3_-treated rats may reflect alterations in neurotrophic support involving both neuronal and glial compartments. These data correlate with a decrease in *Bdnf* expression in patients with AD [49] and presumably might be a consequence of astrocyte hyperreactivity and dysfunction [48]. Furthermore, it has been shown that reduced expression *of Bdnf* and *Ntrk2* induces neuronal shrinkage and loss of function [50], a finding that was also previously demonstrated in our study when assessing the morphological properties of neurons in the hippocampus, primarily in AlCl_3_-treated old rats [21].

MMP-9 is a type IV collagenase that is involved in the degradation of most structural components of the extracellular matrix, leading to both physiological tissue remodeling and pathophysiological changes when the enzyme is overactive [51]. MMP-9 is also a typical and the most well-studied marker of SASP [52]. Consequently, the increase in its concentration in the blood serum of old control animals might occur due to the senescent and pro-inflammatory phenotype of peripheral macrophages—during aging, these cells are the primary contributors to MMP-9 production [53]. However, since aging and microglial activation eventually highly likely lead to a disruption of the blood–brain barrier [54], it is possible that MMP-9 also enters the peripheral bloodstream from the CNS. We have also previously shown that the expression level *of Mmp9*, a marker of microglial activation and aging, did not change in the prefrontal cortex of AlCl_3_-treated adult rats compared to animals in the control group [21]. The increased expression *of Mmp9* in the hippocampus of AlCl_3_-treated adult rats also potentially might be associated with more intensive processing of proBDNF in this brain region to maintain neuroplasticity [55]. Furthermore, the absence of an increase in serum MMP-9 concentration also indirectly indicates the integrity of the blood–brain barrier, since MMP-9 may contribute to an increase in its permeability [56]. Chaperones, including HSP-70, act as a cellular defense mechanism; their expression and secretion increase under stressful conditions [57]. In humans, serum HSP-70 concentrations peak at the age of 25–30 years, and a progressive decline in its level is typically observed in older adults as part of the normal aging process [58]. Oxidative stress and a systemic pro-inflammatory state are interrelated with aging, and HSP-70, as demonstrated in a number of studies, provides protection against the adverse effects of oxidative stress: it restores the conformation of proteins misfolded by excessive ROS activity [22], maintains the stability of GPX4 and prevents its degradation [59], and directly inactivates free radicals [60]. In particular, the extracellular free form of HSP-70, synthesized by various cell, including astrocytes [61], possesses anti-inflammatory properties. In our study, old control animals were not subjected to any stressors under conventional vivarium conditions. Wistar rats also do not have a predisposition to developing age-associated diseases typical for the human population, which is due to species-specific characteristics. Thus, the higher concentration of free HSP-70 in the blood serum of old Wistar rats in the control group compared to adult control animals confirms that these animals possess a phenotype which could be called «healthy aging». Under conditions of oxidative stress, HSP-70 influences both the upregulation of genes encoding antioxidant enzymes, including *Gpx4* [59], as well as their activity [62]; it can also directly scavenge ROS and prevent lipid peroxidation [63]. Thus, the higher serum HSP-70 levels in AlCl_3_-treated adult rats compared to controls also indicate their effective adaptation to stressors. A significant decrease in this adaptation marker protein, combined with the previously identified decrease in *Gpx4* mRNA expression in the prefrontal cortex of AlCl_3_-treated old rats, presumably indicated a lack of resources in these animals to compensate for pro-oxidative processes. It is worth noting that the decrease in serum HSP-70 levels in AlCl_3_-treated old rats also possibly might be associated with the suppression of astrocyte function, since astrocytes are one of the sources of both basal and induced HSP-70 [64]. However, decrease in both MMP-9 and HSP-70 in AlCl_3_-treated rats unlikely directly occurred due to glial changes in the CNS. There was rather co-existence of peripheral markers changes and alterations of microglia and astrocytes than a cause-consequence link. Nevertheless, decreased MMP-9 and HSP-70 potentially indicated impaired adaptive responses. Taken together, these data support the hypothesis that neurodegenerative processes must be modeled in older animals to create pathophysiological conditions that most closely resemble those in older adults.

## 4. Materials and Methods

### 4.1. Animals

This study was conducted on adult (n = 20, age 4–6 months, body weight 420 ± 50 g) and old (n = 20, age 22–24 months, body weight 500 ± 50 g) male Wistar rats (Animal Laboratory Breeding Facility of the Pushchino Branch of the Institute of Biochemistry, Russian Academy of Sciences). Work with experimental animals was conducted in accordance with the principles of the European Convention for the Protection of Vertebrate Animals Used for Experimental Purposes (ETS 123, Strasbourg, 1986) and the Directive of the European Parliament and of the Council of the European Union (2010/63/EU, Strasbourg, 2010). Permission to conduct the experiment was obtained from the Bioethics Committee of the Federal State Budgetary Scientific Institution “Petrovsky National Research Center of Surgery” (Protocol No. 8 dated 29 September 2023). The animals were housed in groups of five per cage under natural lighting, at a temperature of 20–22 °C, with relative humidity of 55–65%, and with free access to water and PK-120-1 compound feed (Laboratorsnab LLC, Certificate of Conformity No. POCCRU.nO81.B00113, GOST P50258-92). The experimental groups of adult (n = 10) and old (n = 10) rats received an aqueous solution of AlCl_3_ at a nominal target dose of 100 mg/kg/day for 60 days via drinking water, as described previously [21]. AlCl_3_ concentration was calculated based on the average body weight of the rats, separately for the groups of adult and old animals. The concentration was 1.9 g AlCl_3_·6H_2_O/L for the adult group and 3.42 g AlCl_3_·6H_2_O/L for the old group. The average daily fluid intake of the rats was determined based on 9–12 mL per 100 g of body weight for animals aged 3–6 months (42 ± 3 mL per day) and 5–7 mL per 100 g of body weight for animals aged 24 months (26 ± 3 mL per day) [65,66]. The volume of solution consumed was measured daily and corresponded to the preliminary calculations of fluid intake by the animals. The daily intake of AlCl_3_ solution as well as body weight of AlCl3-treated adult and old animals did not change along the experiment Animals in the control groups of adult (n = 10) and old (n = 10) rats were given drinking water ad libitum. The groups were formed as follows: Adult-C—control adult rats (n = 10); Adult-AlCl_3_—AlCl_3_-treated adult rats (n = 10); Old-C—control old rats (n = 10); Old-AlCl_3_—AlCl_3_-treated old rats (n = 10). For all further research procedures, samples were divided randomly excluded ELISA of serum samples, where the blood samples of all 10 animals per group were analyzed.

### 4.2. Assessment of Spatial Learning and Memory Using the Morris Water Maze

Spatial orientation and memory functions were assessed on days 52–53 and day 60 of the beginning of the experiment using the Morris water maze. The maze consisted of a circular pool with gray walls, 150 cm in diameter and 60 cm in height, filled with water to a depth of 40 cm. The water temperature was maintained at 22 ± 2 °C. A circular, transparent plastic platform (8 cm in diameter) was submerged 1.5–2 cm below the water surface in the center of one of the pool’s quadrants. The pool was located in a room with fixed spatial cues, and all testing sessions were conducted under natural lighting. The rats underwent one training session per day for two consecutive days. During training, the rats were placed into the water from eight different starting points, positioned at approximately equal distances from the platform and from each other along one semicircle of the pool. Upon reaching the platform, each rat was allowed to remain on it for 15 s before being placed in a holding cage for 60 s. Rats that failed to locate the platform within 60 s were gently guided toward it and also left on the platform for 15 s. For each trial, the time taken to reach the platform was recorded (in seconds). Seven days after the 2nd day of training, a probe trial was conducted: the animal’s movement trajectory was observed for 1 min, and both the latency of crossing the former platform location and the number of such crossings were recorded.

### 4.3. Sample Collection and Preparation

On day 61st of the experiment, blood samples were collected from the neck veins of anesthetized (15 mg/kg of Zoletil (Virbac Sante Animale, Carros, France)) rats; the animals were then euthanized with an overdose (50 mg/kg + 10 mg/kg) of Zoletil + Xylanit (xylazine hydrochloride, 20 mg/mL; NITA-FARM, Saratov, Russia). Death was confirmed by the cessation of spontaneous respiration and heartbeat, together with the absence of corneal reflexes, then the brains were removed. Fragments of the posterior hippocampus (4–6 mm dorsal to bregma, n = 5 from each group) [20] were dissected and fixed in IntactRNA (Eurogen, Moscow, Russia) for further analysis by qPCR-RT and Western blotting. The brain (n = 5 from each group) was fixed in 10% buffered formalin (BioVitrum, Saint-Petersburg, Russia) for 48 h, after which it was dissected at the level of the posterior hippocampus (6 mm dorsal to bregma) [67] and placed in 70° ethanol. The samples were then processed through a series of ascending-concentration ethanol solutions in the Tissue-Tek VIP5Jr automated histological processing system (Sakura, Tokyo, Japan) and embedded in Histomix. Histological sections of 5–7 μm thickness were prepared on a Microm HM340E microtome (Thermo Scientific, Waltham, MA, USA). The research procedures were blinded: A.M.K. coded the samples, while A.V.S., E.A.M., A.D.G., Ch.B.H., and G.M.A. performed IHC, qPCR-RT, and ELISA without knowing which sample belonged to which group.

### 4.4. Immunohistochemical and Morphometric Analysis

Immunofluorescence and immunohistochemical analyses of brain histological sections were performed to identify Iba1+ microglial cells and GFAP+ astrocytes in the hippocampus and EntC. Briefly, histological sections were deparaffinized according to a standard protocol, followed by antigen retrieval in citrate buffer (pH 6.0) containing 0.5% Tween-20 at 100 °C for 20 min. After washing in phosphate-buffered saline (PBS, pH 7.2–7.4), endogenous peroxidase was blocked with a 3% H_2_O_2_ solution for 15 min; non-specific antibody binding was then prevented by blocking with a protein buffer (PBS containing 1% bovine serum albumin and 2% normal goat serum) at room temperature for 30 min. The following primary antibodies were used: anti-Iba1, 1:100 (Rabbit, PAC288Ra01, Cloud-Clone Corp., Katy, TX, US); anti-GFAP, 1:200 (Rabbit, PAA068Ra01, CloudClone, USA); anti-GFAP, 1:500 (Mouse, ab279290, Abcam, Cambridge, UK). Sections were incubated with primary antibodies at +4 °C overnight. The following day, after washing in phosphate-buffered saline, secondary antibody (Caprine-anti-Rabbit HRP, 1:500 (SAA544Rb1, CloudClone, USA), Donkey Anti-Rabbit IgG H&L Texas Red 1:500 (ab6800, Abcam, UK), and Donkey Anti-Mouse Alexa 488 1:500 (718-545-150, Jackson ImmunoResearch Laboratories, Inc. (West Grove, PA, USA)) were applied and incubated at room temperature for 2 h. The HRP labels of the secondary antibodies were visualized using DAB and counterstained with hematoxylin. Fluorescently labeled sections were further visualized using DAPI dye to stain nuclei. On sections stained with the HRP-conjugated secondary antibody, Iba1+ and GFAP+ cells were manually counted on 3 sections per animal (the mean number from the 3 sections was calculated) using the stained sections digitized with a KF-PRO-005 histoscanner (KFBIO, Ningbo, China); cells (n = 50 per group) in CA3 and DG zones of the hippocampus and in layers II, III, and V of EntC were manually outlined, including the soma and the processes, to assess the area. The section area of the hippocampus and EntC was determined using QuPath v.0.6.0 software [68] according to the Atlas of the Rat Brain in stereotaxic coordinates [69].

### 4.5. Western Blotting

To analyze the relative production levels of proteins in the hippocampus samples, Western blot analysis with chemiluminescent detection was used. Initially, five hippocampal samples per experimental group were selected for Western blot analysis. However, one sample from the Adult-AlCl_3_ group and one from the Old-Control group were excluded due to insufficient protein quality, which prevented reliable analysis. Therefore, the final sample sizes were n = 5 for Adult-Control, n = 4 for Adult-AlCl_3_, n = 4 for Old-Control, and n = 5 for Old-AlCl_3_. The exclusion was based on technical quality criteria and was not related to the magnitude or direction of the pTau (Ser404) signal. The fragments were homogenized in RIPA buffer (Sigma Aldrich, St. Louis, MO, USA) with protease inhibitor cocktail (Sigma Aldrich, USA). Then, the homogenate was centrifuged at 14,000 g for 30 min. The supernatant was collected and mixed with the loading buffer containing β-mercaptoethanol, and then heated for 5 min at + 95 °C and stored at −20 °C. Protein separation was carried out by Laemmli electrophoresis in 12.5% polyacrylamide gel. The transfer of proteins to a PVDF membrane (BioRad, Hercules, CA, USA USA) was carried out in Trans-Blot Turbo machine (BioRad, USA) at 25 V for 30 min. To block the sites of nonspecific binding, the membranes were incubated in 5% BSA (InterGen, Oxford, UK) for 1 h at room temperature. Staining with protein-specific antibodies was performed overnight at + 4 °C. The following primary antibodies were utilized in the assay: mouse anti-GAPDH (Cat. № 5G4, clone 4G5, HyTest, Moscow, Russia) (dilution 1:5000), rabbit anti-phospho-Tau (Ser404) (AF3144, Affinity Biosciences, Cincinnati, OH,) (dilution 1:500). After washing, the membranes were placed in a solution of secondary HRP-conjugated antibodies for 1 h at room temperature. The chemiluminescence was visualized with Novex ECL kit (Invitrogen, Carlsbad, CA, USA) on a C-DiGit Blot Scanner (LI-COR, Lincoln, NE, USA) using Image Studio Acquisition Software (version 2.4, LI-COR Biosciences, Lincoln, NE, USA). The sample’s protein content was estimated by normalizing the loading control protein GAPDH.

### 4.6. qPCR-RT

ExtractRNA was used to isolate mRNA from the hippocampus, and reverse transcription was performed using the MMLV RT Kit. Amplification was performed using the qPCRmix-HS SYBR PCR mixture (all from Eurogen, Russia), containing the fluorescent intercalating dye SYBR Green, on a Real-Time DTprime amplifier (DNA-Technology, Moscow, Russia). A single, distinct melting peak was confirmed for each primer pair, confirming amplification specificity and the absence of primer-dimers or non-specific amplification products. Primer amplification efficiency was determined by standard curve analysis and was 98% (E = 1.98; slope = −3.371). We determined the mRNA expression levels of the pro-inflammatory cytokine *Tnf,* the anti-inflammatory cytokine *Tgfb,* the neuroprotective marker *Bdnf* and its receptor *Ntrk2,* the antioxidant enzyme *Gpx4,* and the SASP marker matrix metalloproteinase *Mmp9.* The housekeeping gene *Gapdh* was used as a reference since it was described as reliable when assessing gene expression in brain of adult and old rats [70]. In all samples in the control and experimental groups, *Gapdh* expression was recorded at 21–22 cycles; thus, it was proved stable. For gene expression analysis, the threshold cycle (Ct) method was employed, and relative gene expression was calculated using the method proposed by Pfaffl M.W. [71]. Each biological sample (n = 5 per group) has three technical replicates, and their mean Ct values were used for calculation. The relative mRNA concentrations of the specified genes were determined via direct data comparison using the formula [A]0/[B]0 = EΔCt, where [A]0 represents the initial mRNA concentration of the target gene in the PCR mixture; [B]0 represents the initial mRNA concentration of GAPDH in the PCR mixture; E denotes the reaction efficiency (1.98); and ΔCt represents the difference between the threshold cycles of GAPDH and the target gene, calculated using Microsoft Office Excel 2016. For gene expression analysis, the cycle threshold (ΔCt) method was used to determine relative gene expression. Primers were selected using Primer-BLAST software (Primer3 version 2.5.0, NCBI, Bethesda, MD, USA) (species-specific for *Rattus norvegicus*). All primer pairs were designed and optimized to ensure comparable amplification performance under standardized RT-qPCR conditions. Amplification efficiencies was evaluated during previous methodological validation and were found to be comparable among the selected primer pairs. Therefore, identical amplification conditions were used for all target and reference genes. The primer sequences are presented in Table 2.

### 4.7. ELISA

Using the enzyme-linked immunosorbent assay (ELISA) method, the levels of free HSP-70 chaperone (FineTest, Wuhan, China) and matrix metalloproteinase MMP-9 (FineTest, China) were determined in the blood serum of rats from the control and experimental groups (n = 10 per group). The level of 4-hydroxy-2-onenal (4-HNE) was assessed in the supernatant of the hippocampus fragments homogenate (n = 5 per group) using ELISA kit (Elabscience, Wuhan, China). An AMR-100 microplate reader (Allsheng, Hangzhou, China) and commercial kits were used in accordance with the manufacturer’s instructions.

### 4.8. Statistical Analysis

Statistical analyses were performed using GraphPad Prism version 9.1 (GraphPad Software, San-Diego, CA, USA) and R software (version 4.6.1; R Foundation for Statistical Computing, Vienna, Austria). Data normality was assessed using the Shapiro–Wilk test. Since the data were not normally distributed, nonparametric statistical methods were applied. Comparisons among multiple groups were performed using the Scheirer–Ray–Hare test, including the calculation of size effect, followed by Dunn’s post hoc test with Benjamini–Hochberg correction for multiple comparisons. For each experimental endpoint, four biologically relevant pairwise comparisons were predefined: (1) Adult-Control vs. Adult-AlCl_3_, (2) Old-Control vs. Old-AlCl_3_, (3) Adult-Control vs. Old-Control, and (4) Adult-AlCl_3_ vs. Old-AlCl_3_. Dunn’s post hoc test was performed for these comparisons, and the resulting *p*-values were adjusted using the Benjamini–Hochberg procedure. The proportion of animals that crossed the former platform location during the probe trial was compared using Fisher’s exact test. The latency to the first crossing of the former platform location during the 60-s probe trial was analyzed using the Kaplan–Meier method and the log-rank test with Benjamini–Hochberg correction. Animals that did not reach the former platform location within the observation period were treated as right-censored at 60 s. Overall differences among the four experimental groups were assessed using the log-rank test, followed by pairwise log-rank comparisons with Benjamini–Hochberg correction. Data were presented as the median (Me) with the interquartile range (25%; 75%). Differences were considered statistically significant at *p* <0.05.

## 5. Limitations

Several limitations of this study should be considered when interpreting the findings. The study was exploratory and aimed primarily at identifying age- and brain-region-specific patterns of glial responses to chronic oxidative stress rather than providing confirmatory evidence for individual molecular mechanisms. Although the initial experimental groups included 10 animals each, histological and RT-qPCR analyses were performed using separate subsets of 5 animals per group. The relatively small sample size may have limited statistical power, particularly for endpoints characterized by substantial interindividual variability such as GFAP+ astrocyte number. Therefore, non-significant findings should not necessarily be interpreted as evidence for the absence of biologically relevant effects, and the identified patterns require confirmation in larger independent cohorts.

The study included only male Wistar rats. Sex differences have been reported in aging, pro-inflammatory responses, oxidative stress, and glial function; therefore, the present findings cannot be directly generalized to females. Future studies incorporating both sexes are warranted to determine whether the age-dependent glial responses observed in this model are sex-specific.

Western blot analysis was limited by the relatively small sample size, with four to five biological samples per group, and by the assessment of only one Tau phosphorylation site (Ser404) normalized to GAPDH, whereas total Tau abundance was not measured. Therefore, the observed differences in pTau (Ser404)/GAPDH values cannot be unambiguously attributed to changes in Tau phosphorylation. Furthermore, although a significant age-associated difference was detected, neither the main effect of AlCl_3_ treatment nor the age × treatment interaction reached statistical significance. Accordingly, these findings should be regarded as exploratory observations rather than evidence of AlCl_3_-induced Tau hyperphosphorylation. Future studies should include larger cohorts, total Tau measurements, and additional phosphorylation sites.

Glial morphology was assessed using GFAP and Iba1 immunoreactivity, cell density, cell area, and qualitative morphological observations. Although these methods revealed age- and region-related structural alterations, they do not permit unequivocal identification of reactive, dystrophic, senescent, or functionally impaired glial states. In particular, changes in cell morphology cannot independently establish reduced neuroprotective activity or neuronal dysfunction. Additional cell-specific molecular markers, more detailed morphometric analyses, and functional assays will be required to determine the biological significance of these observations. Molecular analyses were restricted to the hippocampus and a panel of selected genes. Gene expression does not necessarily reflect corresponding protein abundance or biological activity, and the molecular mechanisms underlying the pronounced region-specific glial alterations, particularly those observed in the entorhinal cortex, therefore remain to be established. Future studies should include molecular analyses of both brain regions and complementary protein-level and functional assays.

Finally, serum MMP-9 and HSP-70 were investigated as exploratory systemic markers associated with inflammation, cellular stress, and adaptive responses. Their circulating concentrations are influenced by multiple peripheral tissues and cannot be considered specific indicators of CNS processes. The present study therefore does not establish a direct mechanistic relationship between serum MMP-9 or HSP-70 concentrations and the observed glial alterations in the brain. Their potential value as peripheral correlates of age-dependent responses to oxidative stress should be validated by simultaneous assessment of these proteins in brain tissue and serum and, where appropriate, by evaluating blood–brain barrier integrity.

Taken together, these limitations emphasize the hypothesis-generating nature of the present study. Nevertheless, the combination of two age groups, two brain regions, and complementary morphological, molecular, and systemic measurements provides a framework for identifying age- and region-dependent patterns of glial vulnerability to chronic oxidative stress that can be tested in subsequent adequately powered mechanistic studies.

## 6. Conclusions

The present exploratory study demonstrated that aging is associated with region-specific morphological and molecular responses to chronic AlCl_3_ exposure in male Wistar rats. Aging was accompanied by increased Iba1+ microglial and GFAP+ astrocytic cell area in the hippocampus and entorhinal cortex, together with increased hippocampal *Tnf* expression and reduced *Tgfb* and *Ntrk2* expression. These findings were accompanied by co-existing age-related differences in circulating MMP-9 and HSP-70 concentrations.

Chronic AlCl_3_ exposure was associated with distinct glial morphological alterations depending on age and brain region. In adult rats, these changes primarily involved increased Iba1+ cell density in the entorhinal cortex and alterations in astrocytic morphology. In old rats, AlCl_3_ exposure was associated with increased Iba1+ cell density in both investigated regions, altered GFAP+ astrocytic morphology, and reduced astrocytic cell area in the entorhinal cortex. No significant treatment-associated increase in hippocampal GFAP+ cell density was detected.

Old AlCl_3_-treated animals also demonstrated reduced success in locating the former platform position during the Morris water maze probe trial and increased hippocampal 4-HNE levels, accompanied by reduced *Bdnf* and *Gpx4* expression. Although an age-related difference in hippocampal pTau (Ser404) immunoreactivity was observed, no significant effect of AlCl_3_ treatment or age × treatment interaction was detected.

Overall, the findings identify age- and region-dependent patterns of glial morphological alterations and molecular responses under chronic oxidative stress. These results provide a basis for further investigation of the relationships among aging, oxidative stress, glial responses, and neurodegeneration, while additional functional and neuropathological studies are needed to establish the underlying mechanisms and their relevance to translational modeling of AD.

## Figures and Tables

**Figure 1:**
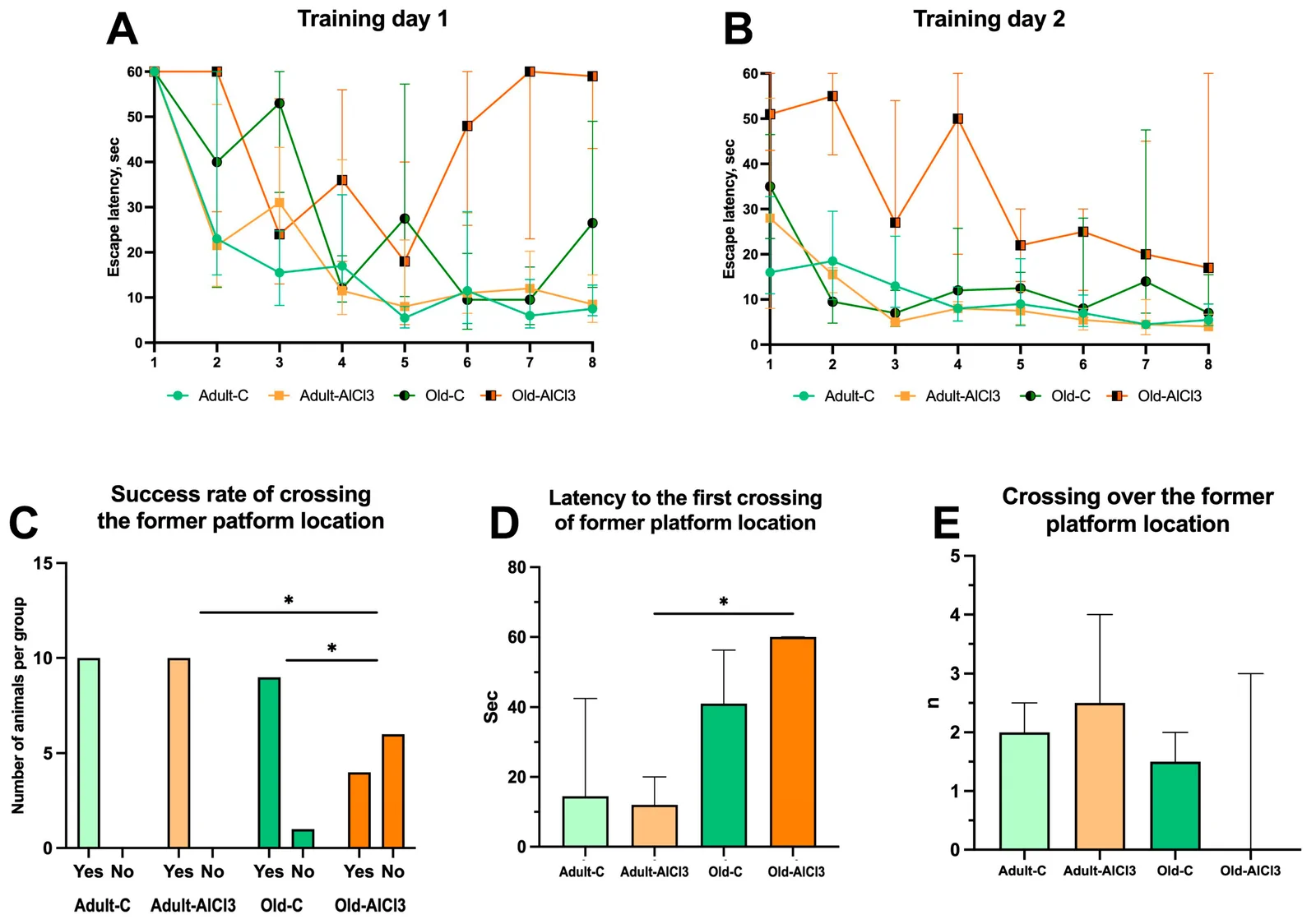
Assessment of spatial learning and memory using the Morris water maze in adult and old male Wistar rats following prolonged AlCl_3_ administration. (**A,B**) Escape latency (s) during eight consecutive trials on training days 1 (**A**) and 2 (**B**). (**C**) Number of animals that successfully crossed (Yes) or did not cross (No) the former platform location during the probe trial conducted seven days after training. (**D**) Latency to the first crossing of the former platform location during the 60-s probe trial. Animals that did not cross the target location were assigned a latency of 60 s and treated as right-censored observations in Kaplan–Meier analysis. (**E**) Number of crossings over the former platform location during the probe trial. Adult-C—control adult rats (*n* = 10); Adult-AlCl_3_—AlCl_3_-treated adult rats (*n* = 10); Old-C—control old rats (*n* = 10); Old-AlCl_3_—AlCl_3_-treated old rats (*n* = 10). *—*p* < 0.05. Multiple comparisons were performed using the nonparametric Scheirer–Ray–Hare test, followed by Dunn’s post hoc test with Benjamini–Hochberg correction. Data are presented as Me (LQ 25%; UQ 75%) (**A**,**B**,**D**,**E**) and absolute numbers of animals (**C**). Differences in crossing success rates were assessed using Fisher’s exact test.

**Figure 2:**
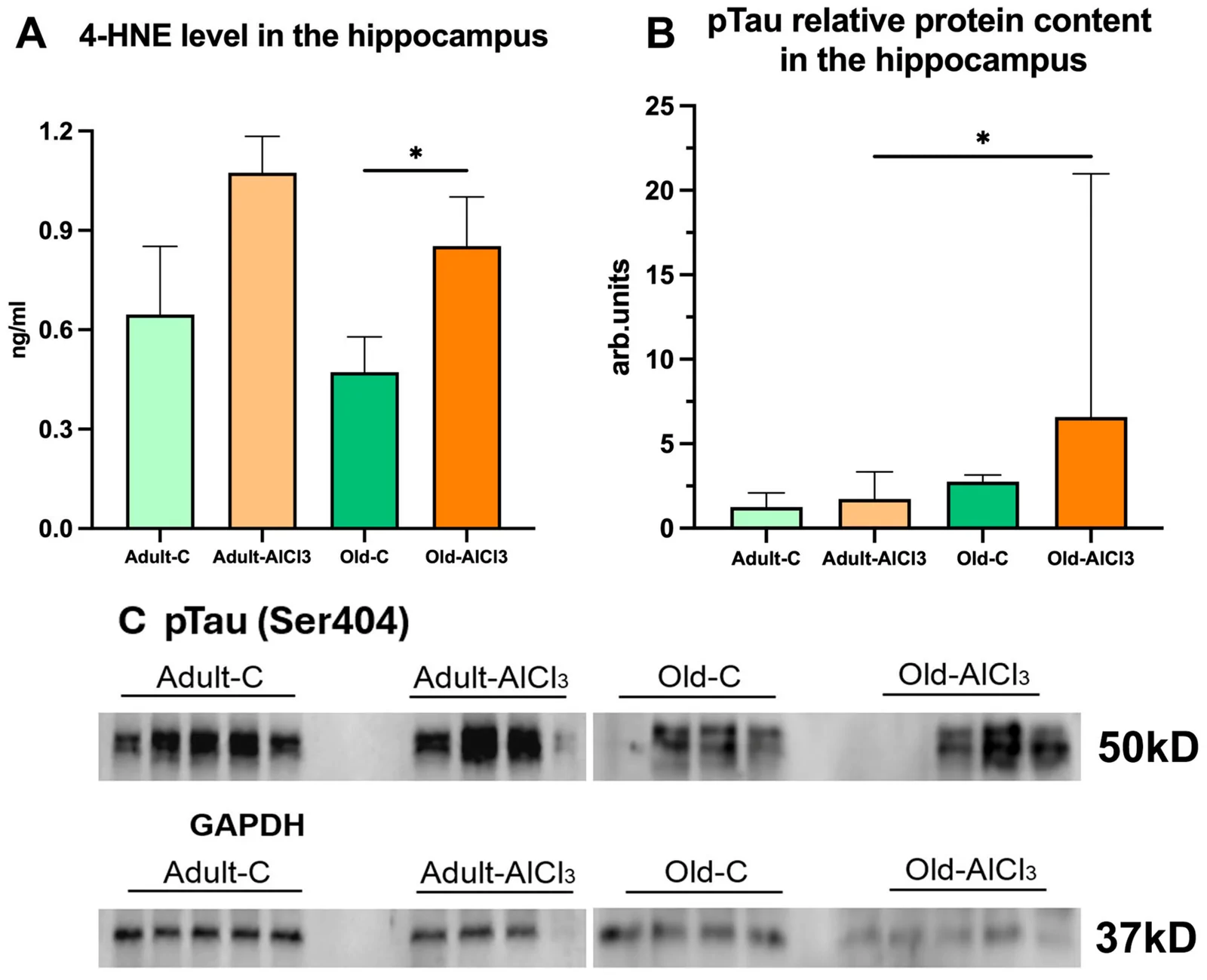
(**A**) The level of 4-HNE (**A**) in the hippocampus of adult and old male Wistar rats following prolonged AlCl_3_ administration. Adult-C—control adult rats (*n* = 5); Adult-AlCl_3_—AlCl_3_-treated adult rats (*n* = 5); Old-C—control old rats (*n* = 5); Old-AlCl_3_—AlCl_3_-treated old rats (*n* = 5). (**B**,**C**) GAPDH-normalized pTau (Ser404) immunoreactivity (**B**,**C**) in the hippocampus of adult and old male Wistar rats following prolonged AlCl_3_ administration. Adult-C—control adult rats (*n* = 5); Adult-AlCl_3_—AlCl_3_-treated adult rats (*n* = 4); Old-C—control old rats (*n* = 4); Old-AlCl_3_—AlCl_3_-treated old rats (*n* = 5). *—*p* < 0.05. Multiple comparisons were performed using the nonparametric Scheirer–Ray–Hare test, followed by Dunn’s post hoc test with Benjamini–Hochberg correction. Data are presented as Me (LQ 25%; UQ 75%).

**Figure 3:**
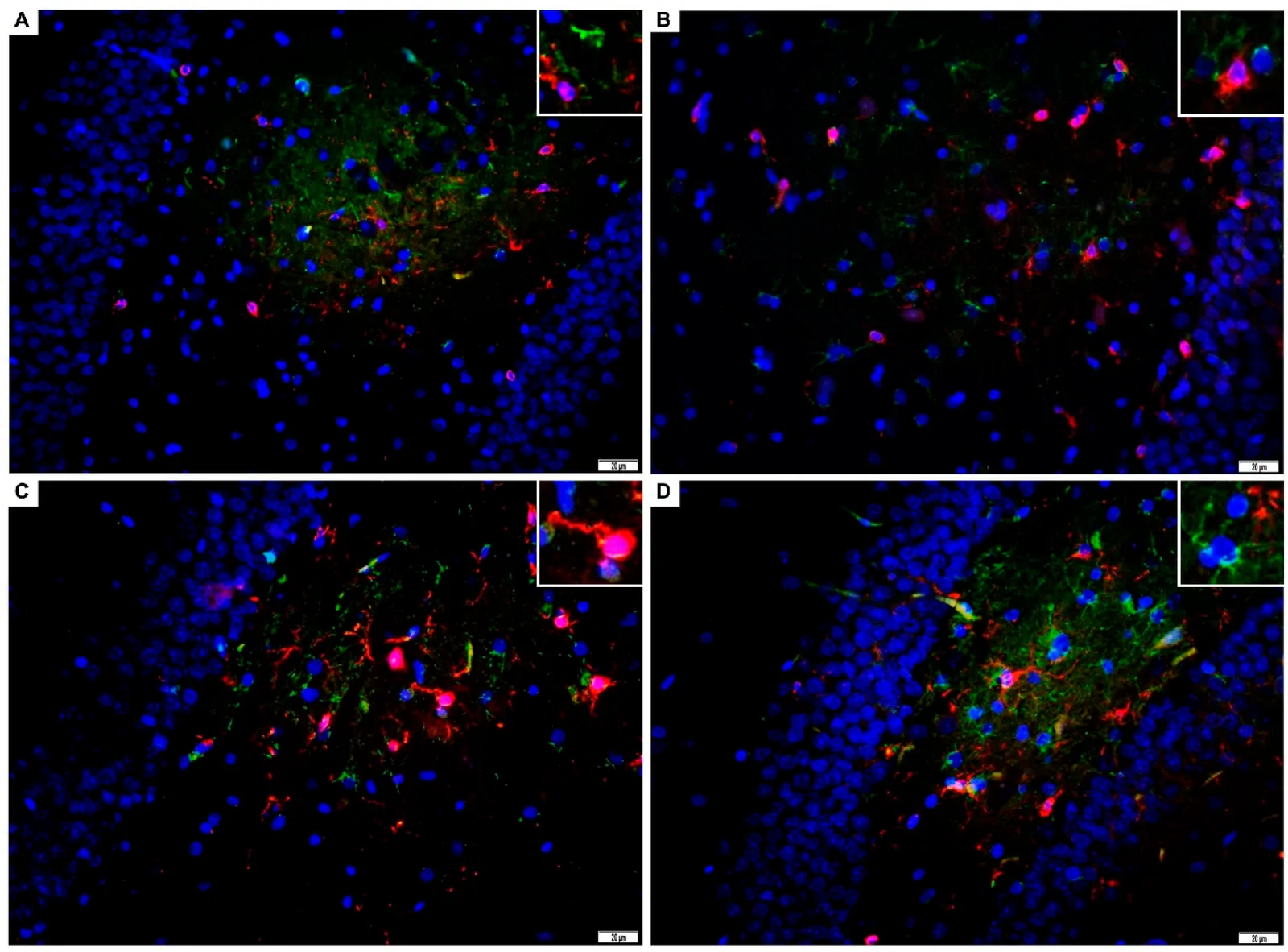
Colocalization of GFAP+ astrocytes (green fluorescence) and Iba1+ microglial cells (red fluorescence) in the hippocampus of adult and old male Wistar rats following prolonged AlCl_3_ administration. (**A**) Adult-C: Small GFAP+ astrocytes and Iba1+ microglial cells with few thin processes; (**B**) Adult-AlCl_3_: Small GFAP+ astrocytes and Iba1+ microglial cells with few thin processes; (**C**) Old-C: Enlarged GFAP+ astrocytes (>20–30 μm) with thickened processes and enlarged Iba1+ microglial cells (>20–30 μm) with thickened, branched processes; (**D**) Old-AlCl_3_: enlarged GFAP+ astrocytes (>20–30 μm) with numerous branched and hypertrophied processes and enlarged Iba1+ microglial cells (>20–30 μm) with thickened processes. anti-GFAP + Alexa Fluor 488, anti-Iba1 + Alexa Fluor 594 + DAPI, ×400. Scale bar: 20 μm.

**Figure 4:**
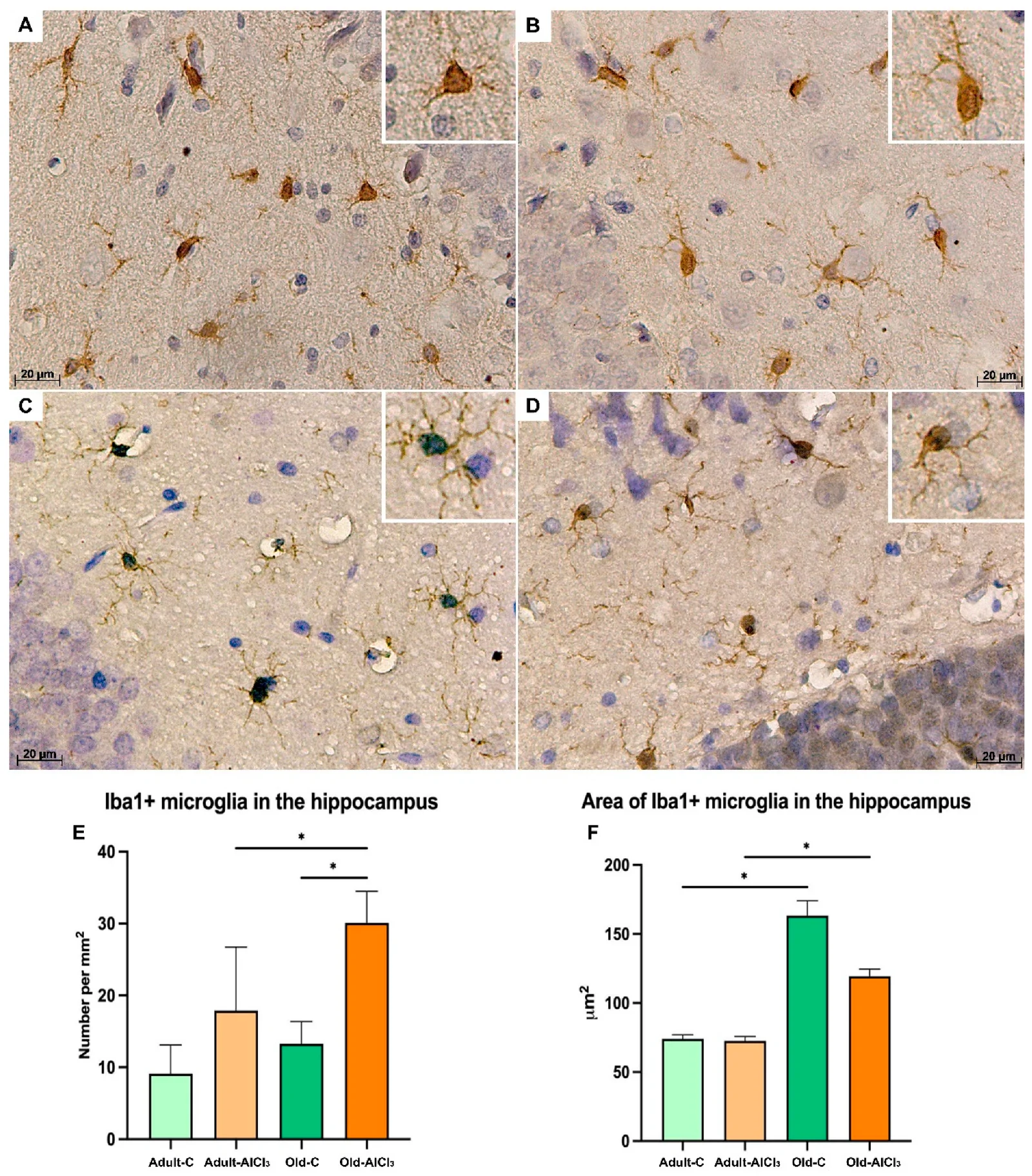
Iba1+ microglia in the hippocampus of adult and old male Wistar rats following prolonged AlCl_3_ administration. (**A**) Adult-C: a small number of Iba1+ microglial cells with moderately branched, thin processes; (**B**) Adult-AlCl_3_: a moderately higher number of Iba1+ microglial cells with more branched processes; (**C**) Old-C: enlarged Iba1+ microglia (>20–30 μm) with enlarged branched processes; (**D**) Old-AlCl_3_: enlarged Iba1+ microglia (>20–30 μm) with less numerous and branched processes. anti-Iba1 + HRP, counterstained with hematoxylin, ×640. (**E**) The absolute number of Iba1+ cells in the hippocampus per mm^2^ in adult and old rats from the control and AlCl_3_-treated groups. (**F**) The area of Iba1+ cells in the hippocampus of adult and old rats in the control and AlCl_3_-treated groups. Adult-C—control adult rats (n = 5); Adult-AlCl_3_—AlCl_3_-treated adult rats (n = 5); Old-C—control old rats (n = 5); Old-AlCl_3_—AlCl_3_-treated old rats (n = 5). *—*p* < 0.05. Multiple comparisons were performed using the nonparametric Scheirer–Ray–Hare test, followed by Dunn’s post hoc test with Benjamini–Hochberg correction. Data are presented as Me (LQ 25%; UQ 75%).

**Figure 5:**
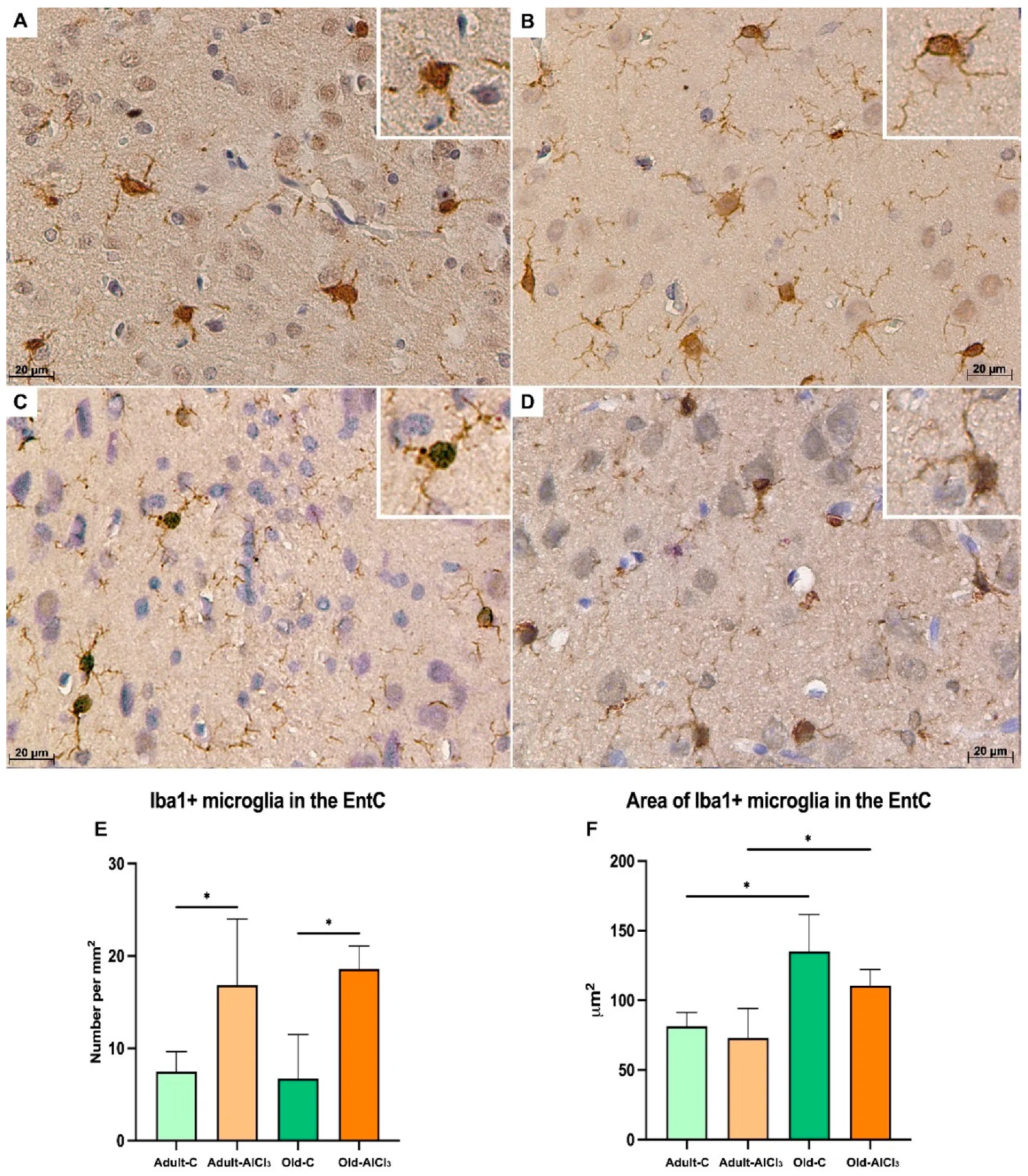
Iba1+ microglia in the EntC of adult and old male Wistar rats following prolonged AlCl_3_ administration. (**A**) Adult-C: a small number of Iba1+ microglial cells with moderately branched, thin processes; (**B**) Adult-AlCl_3_: a higher number of Iba1+ microglial cells with more branched processes; (**C**) Old-C: enlarged Iba1+ microglia (>20–30 μm) with enlarged hypertrophic processes; (**D**) Old-AlCl_3_: a higher number of enlarged Iba1+ microglia (>20–30 μm) with less hypertrophic processes. anti-Iba1 + HRP, counterstained with hematoxylin, ×640. (**E**) The absolute number of Iba1+ cells in the EntC per mm^2^ in adult and old rats from the control and AlCl_3_-treated groups. (**F**) The area of Iba1+ cells in the EntC of adult and old rats in the control and AlCl_3_-treated groups. Adult-C—control adult rats (n = 5); Adult-AlCl_3_—AlCl_3_-treated adult rats (n = 5); Old-C—control old rats (n = 5); Old-AlCl_3_—AlCl_3_-treated old rats (n = 5). *—*p* < 0.05. Multiple comparisons were performed using the nonparametric Scheirer–Ray–Hare test, followed by Dunn’s post hoc test with Benjamini–Hochberg correction. Data are presented as Me (LQ 25%; UQ 75%).

**Figure 6:**
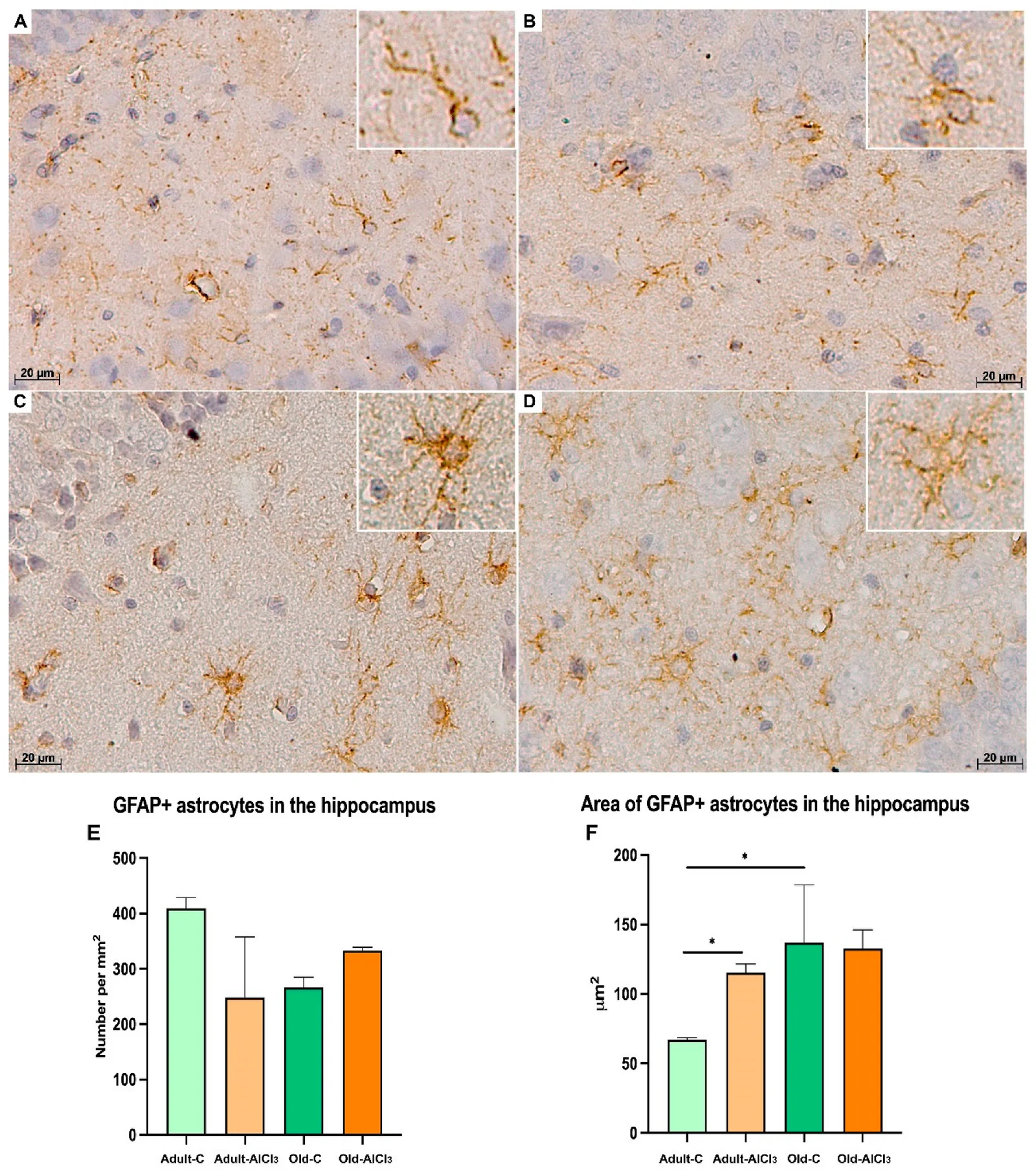
GFAP+ astrocytes in the hippocampus of adult and old male Wistar rats following prolonged AlCl_3_ administration. (**A**) Adult-C: a small number of small GFAP+ astrocytes with moderately branched, thin processes; (**B**) Adult-AlCl_3_: a small number of small GFAP+ astrocytes with more branched processes; (**C**) Old-C: enlarged GFAP+ astrocytes (>20–30 μm) with numerous branched processes; (**D**) Old-AlCl_3_: enlarged astrocytes (>20–30 μm) with numerous branched and hypertrophied processes. anti-GFAP + HRP, counterstained with hematoxylin, ×640. (**E**) The absolute number of GFAP+ cells in the hippocampus per mm^2^ in adult and old rats from the control and AlCl_3_-treated groups. (**F**) The area of GFAP+ cells in the hippocampus of adult and old rats in the control and AlCl_3_-treated groups. Adult-C—control adult rats (n = 5); Adult-AlCl_3_—AlCl_3_-treated adult rats (n = 5); Old-C—control old rats (n = 5); Old-AlCl_3_—AlCl_3_-treated old rats AlCl_3_ (n = 5). *—*p* < 0.05. Multiple comparisons were performed using the nonparametric Scheirer–Ray–Hare test, followed by Dunn’s post hoc test with Benjamini–Hochberg correction. Data are presented as Me (LQ 25%; UQ 75%).

**Figure 7:**
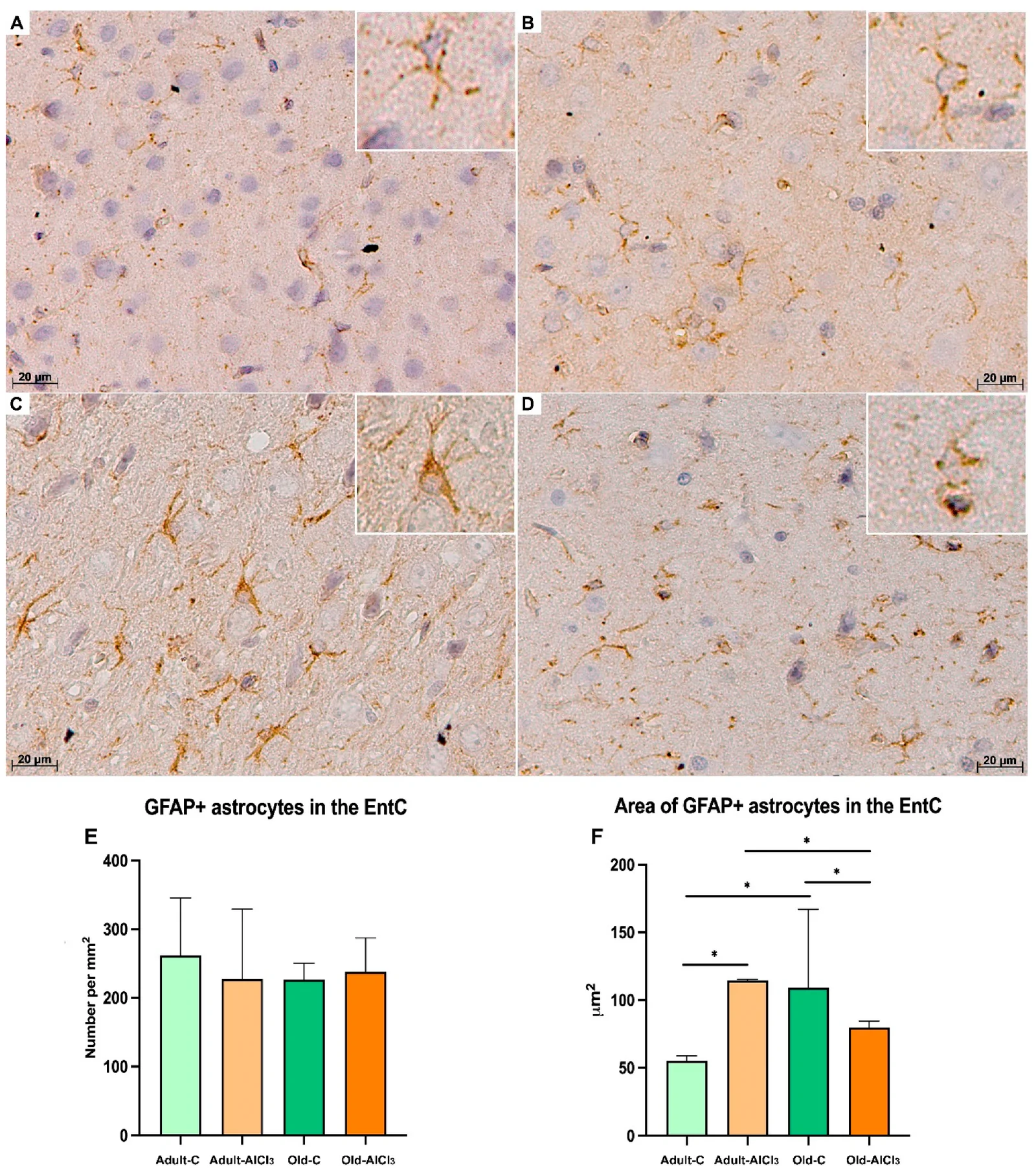
GFAP+ astrocytes in the EntC of adult and old male Wistar rats following prolonged AlCl_3_ administration. (**A**) Adult-C: a small number of small GFAP+ astrocytes with moderately branched, thin processes; (**B**) Adult-AlCl_3_: a small number of small GFAP+ astrocytes with more branched processes; (**C**) Old-C: enlarged GFAP+ astrocytes (>20–30 μm) with a small number of thickened processes; (**D**) Old-AlCl_3_: small astrocytes (10–20 μm) with thinned, fragmented processes. anti-GFAP + HRP, counterstained with hematoxylin, ×640. (**E**) The absolute number of GFAP+ cells in the EntC per mm^2^ in adult and old rats from the control and AlCl_3_-treated groups. (**F**) The area of GFAP+ cells in the EntC of adult and old rats in the control and AlCl_3_-treated groups. Adult-C—control adult rats (n = 5); Adult-AlCl_3_—AlCl_3_-treated adult rats (n = 5); Old-C—control old rats (n = 5); Old-AlCl_3_—AlCl_3_-treated old rats AlCl_3_(n = 5). *—*p* < 0.05. Multiple comparisons were performed using the nonparametric Scheirer–Ray–Hare test, followed by Dunn’s post hoc test with Benjamini–Hochberg correction. Data are presented as Me (LQ 25%; UQ 75%).

**Figure 8:**
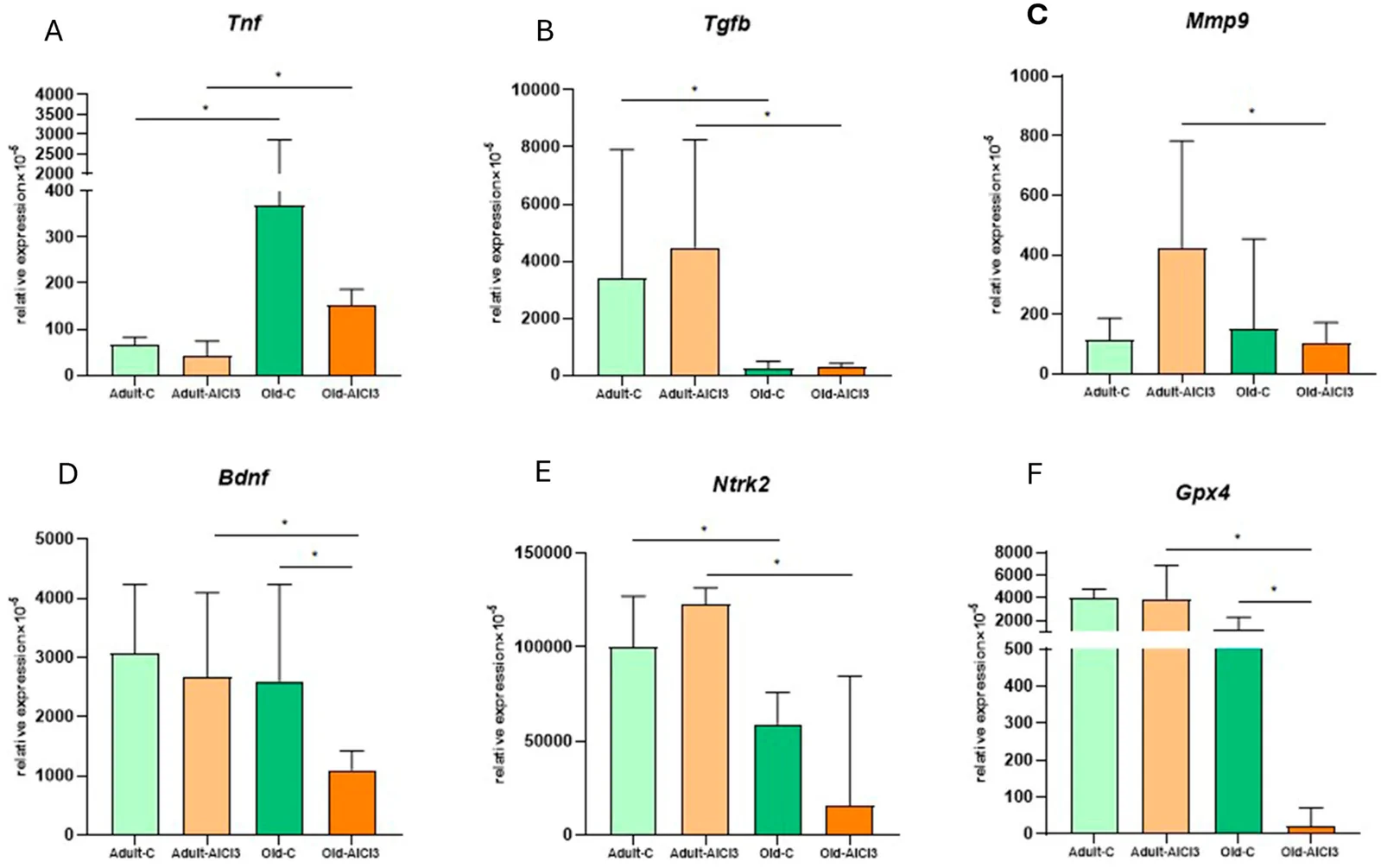
mRNA expression *of Tnf* (**A**), *Tgfb* (**B**), *Mmp9* (**C**), *Bdnf* (**D**), *Ntrk2* (**E**), and *Gpx4* (**F**) in the hippocampus of adult and old male Wistar rats following prolonged AlCl_3_ administration. Adult-C— control adult rats (n = 5); Adult-AlCl_3_—AlCl_3_-treated adult (n = 5); Old-C—control old rats (n = 5); Old-AlCl_3_— AlCl_3_-treated old rats (n = 5). *—*p* < 0.05. Multiple comparisons were performed using the nonparametric Scheirer–Ray–Hare test, followed by Dunn’s post hoc test with Benjamini–Hochberg correction. Data are presented as Me (LQ 25%; UQ 75%).

**Figure 9:**
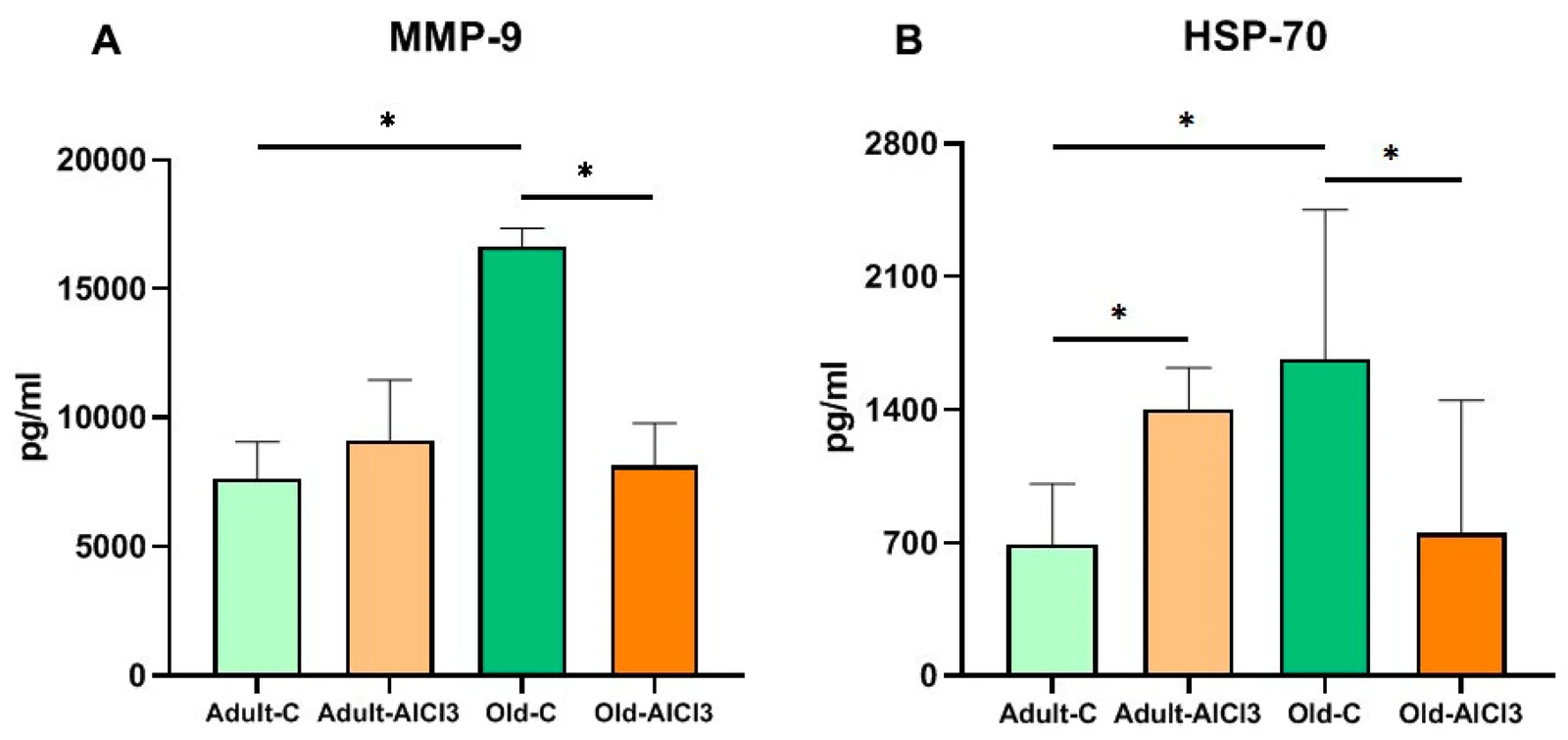
Serum levels of MMP-9 (**A**) and HSP-70 (**B**) in adult and old male Wistar rats following prolonged AlCl_3_ administration. Adult-C—control adult rats (n = 10); Adult-AlCl_3_—AlCl_3_-treated adult rats (n = 10); Old-C—control old rats (n = 10); Old-AlCl_3_—AlCl_3_-treated old rats (n = 10). *—*p* < 0.05. Multiple comparisons were performed using the nonparametric Scheirer–Ray–Hare test, followed by Dunn’s post hoc test with Benjamini–Hochberg correction. Data are presented as Me (LQ 25%; UQ 75%).

**Table 1:** Size effect of age, AlCl_3_ exposure and Age × AlCl_3_ exposure of Iba1+ microglia and GFAP+ astrocytes number and area in the hippocampus and the EntC of adult and old male Wistar rats following prolonged AlCl_3_ administration.

Comparison Factor	Iba1+ Microglia				GFAP+ Astrocytes			
	EntC		Hippocampus		EntC		Hippocampus	
	Number	Area	Number	Area	Number	Area	Number	Area
Age	0.274; 0.601; 0.009	**21.899; <0.0001; 0.562**	**6.981; 0.008; 0.233**	**23.273; <0.0001; 0.751**	0.007; 0.935; <0.001	1.548; 0.213; 0.040	0.149; 0.700; 0.009	**19.441; <0.0001; 0.498**
AlCl_3_ exposure	**18.210; <0.0001; 0.628**	1.979; 0.160; 0.051	**10.370; 0.001; 0.346**	1.945; 0.163; 0.063	0.027; 0.870; 0.001	1.979; 0.160; 0.051	0.149; 0.700; 0.009	2.134; 0.144; 0.055
Age × AlCl_3_ exposure	0.073; 0.786; 0.003	1.113; 0.291; 0.029	0.119; 0.730; 0.004	0.513; 0.474; 0.017	0.788; 0.375; 0.044	**29.268; <0.0001; 0.750**	**4.542; 0.033; 0.284**	**4.801; 0.028; 0.123**

Data represented as H, Scheirer–Ray–Hare test statistic; *p*, *p*-value; η^2^H, effect size estimate for the Scheirer–Ray–Hare test.

**Table 2:** Primer sequences used in qPCR-RT analysis.

Encoded Gene	Forward Primer Sequence (5′→3′)	Reverse Primer Sequence (5′→3′)
*Bdnf*	CGAAGAGCTGCTGGATGAG	ATGGGATTACACTTGGTCTCG
*Ntrk2*	CACACACAGGGCTCCTTA	AGTGGTGGTCTGAGGTTGG
*Gpx4*	AATTCGCAGCCAAGGACATCG	ATTCGTAAACCACACTCGGCGTA
*Mmp9*	ATGGTTTCTGCCCCAGTGAG	CACCAGCGATAACCATCCGA
*Tnf*	CCACCACGCTCTTCTGTCTA	GCTACGGGCTTGTCACTCG
*Tgfb*	CCGCAACAACGCAATCTATG	AGCCCTGTATTCCGTCTCCTT
*Gapdh*	GCGAGATCCCGCTAACATCA	CCCTTCCACGATGCCAAAGT

## Data Availability

All data analyzed during this study are included in this article and available from the corresponding author upon reasonable request.
